# Long-term integrative treatment of bipolar disorder

**DOI:** 10.1192/j.eurpsy.2026.10767

**Published:** 2026-07-31

**Authors:** L. Mehl-Madrona, B. J. Mainguy

**Affiliations:** 1 Psychiatry Residency, Northern Light Health, Bangor; 2 Family Medicine, University of New England, Portland; 3 Family Medicine Residency, Northern Light Health, Bangor; 4 Research, Coyote Institute; 5 Philosophy, University of Maine; 6 Psychotherapy, Maine Street Medicine Works, LLC; 7 Education, Coyote Institute, Orono, United States

## Abstract

**Introduction:**

While double-blinded, randomized controlled trials are considered the gold standard for assessing therapies, they are not always practical when therapies last for years with complicated patients for whom controls would be difficult to find. A rationale exists for looking at the effects of real-world practice where RCTs are impractical and too expensive.

**Objectives:**

We have been working in the field of Inegrative Psychiatry since 1990, providing long-term management of bipolar disorder with a goal to eliminate or reduce medications. We wanted to present the results we have accumulated over a 35 year period to show what is possible.

**Methods:**

The setting was a private practice which provided free or reduced fee care for Indigenous people. Our approach falls into three arms: full integrative management, multi-nutrients and nutrition alone with or without psychotherapy, and conventional care with medication minimization, also with or without psychotherapy. No patient was turned away. We used the clinical global impressions (CGI) scale with all patients along with the Young Mania Scale (YMS) and the Montgomery-Asburg Depression Scale (MADRS).

**Results:**

To date we have seen 307 patients for an average of 18.7 months. Seventy-two people engaged in full integrated care consisting of psychotherapy, mindfulness meditation, acupuncture, osteopathy, yoga, nutritional modifications and micronutrients, and physical exercise for an average of 26.4 months. Sixty-nine of those patients were able to eliminate conventional medications. Fifty-nine patients participated in micronutrient supplementation alongside conventional medication for 15.3 months. Fifty four of those patients were able to reduce or eliminate conventional medication. Two hundred seventy two patients participated in conventional care with an aim to reduce medications. Twenty seven were able to eliminate medication and 120 were able to reduce medications. The remainder maintained (20) or increased (55) their use of medications over time. This group stayed for an average of 161.5 months. Successful strategies are multi-modal, supported by family and community members with feedback at early signs of mania or returning depression, and required continued, diligent application of practices. Unsuccessful approaches tended to involve only one or two modalities, tended to lack family or community support,and lacked early warning systems, Patients appeared equally severe at intake based on number of prior hospitalizations, number of prior suicide attempts, medication equivalencies, and similar scores on the CGI, MADRS, and the YMS. Statistically significant improvement occurred on the CGI, YMS, and MADRS for all patients.

**Conclusions:**

Integrative psychiatric treatment over time can help people function without psychiatric medications but commitment is required at a level not all patients can reach or afford. New research paradigms are needed that address long-term treatments for complex problems.

**Disclosure of Interest:**

None Declared

